# MS-DASPNet: Multiple Sclerosis lesion segmentation from brain MRI using dual attention and spatial pyramid pooling with transfer learning

**DOI:** 10.3389/fncom.2025.1713766

**Published:** 2026-01-29

**Authors:** Shikha Jain, Navin Rajpal, Pramod Kumar Soni

**Affiliations:** 1 University School of Information, Communication & Technology, Guru Gobind Singh Indraprastha University, New Delhi, India; 2 Department of Computer Applications, Manipal University Jaipur, Jaipur, India

**Keywords:** atrous spatial pyramid pooling, CNN, deep learning, double-headed attention, multiple sclerosis

## Abstract

Accurate detection and segmentation of multiple sclerosis (MS) lesions in brain Magnetic Resonance Imaging (MRI) is a challenging task due to their small size, irregular shape, and variability in different imaging modalities. Precise segmentation of MS lesions from brain MRI is vital for early diagnosis, disease progression monitoring, and treatment planning. We introduce MS-DASPNet, a Dual Attention Guided Deep Neural Network specifically designed to address the challenges of MS lesion detection, including small lesion sizes, low contrast, and heterogeneous appearance. MS-DASPNet employs a VGG-16-based encoder, an Atrous Spatial Pyramid Pooling (ASPP) bottleneck for multi-scale context learning, and dual attention modules in each skip connection to simultaneously refine spatial details and enhance channel-wise feature representation. Evaluations on four publicly available datasets, namely ISBI-2015, Mendeley, MICCAI-2016, and MICCAI-2021, demonstrate that MS-DASPNet achieves superior Precision, Dice, Sensitivity, and Jaccard scores compared to state-of-the-art methods. MS-DASPNet attains a Dice score of 0.8736 on the MICCAI-2016 dataset and 0.8706 on the MICCAI-2021 dataset, both outperforming existing segmentation techniques, highlighting its robustness and effectiveness in accurate MS lesion segmentation.

## 1 Introduction

Multiple sclerosis (MS) is a long-term autoimmune disease that targets the central nervous system (CNS), characterized by damage to the myelin sheath, degeneration of nerve fibers, and inflammation within neural tissues (Compston and Coles, 2008). The disease disrupts communication between the brain and body, causing motor, sensory, and cognitive impairments. MS lesions develop in different brain regions, forming sclerosis that appears in multiple locations, thus giving the disease its name, MS (Filippi et al., 2016). While the exact origin of MS remains unclear, genetic predisposition, environmental factors, infections, and immune system dysfunction are believed to contribute to disease onset (Olsson et al., 2017). Magnetic Resonance Imaging (MRI) is the most effective imaging modality for detecting MS lesions, as it provides detailed visualization of white matter abnormalities across multiple sequences, including T1-weighted, T2-weighted, Fluid-Attenuated Inversion Recovery (FLAIR), and Proton Density (PD) scans (Zivadinov et al., 2008). However, accurately segmenting MS lesions remains a difficult task due to variability in lesion appearance, image artifacts, and the complexity of brain anatomy. Various approaches, including traditional techniques, machine learning, and DL methods, have been investigated for MS lesion segmentation. These approaches are generally classified into supervised, unsupervised, and DL-based categories.

Supervised machine learning (ML) techniques require labeled training data, where models learn to distinguish lesions from healthy tissue. Traditional approaches such as thresholding, region growth, and statistical models (e.g., Gaussian Mixture Models and Bayesian classifiers) were initially applied to segmentation (Sajja et al., 2006; Anbeek et al., 2005). Later, advanced classifiers such as Support Vector Machines (SVMs), the Hidden Markov Model, and the expectation-maximization algorithm demonstrated improved lesion detection (Zhang et al., 2001; Lao et al., 2008). More recently, supervised learning models have leveraged hand-crafted features, including intensity histograms, texture features, and spatial priors, to refine segmentation accuracy (Jain et al., 2020). However, supervised models often require extensive manual annotation, which is labor-intensive and prone to inter-rater variability.

Unsupervised algorithms identify hidden patterns in data to facilitate classification and segmentation tasks. Various unsupervised techniques have been explored for MS lesion segmentation. A feature vector-based approach has been utilized to segment MS lesions from skull-stripped MRI images (Akbarpour et al., 2017). Robust partial-volume tissue segmentation, which integrates intensity-based probabilistic and morphological prior maps, incorporating outlier rejection and filling, has also been proposed (Valverde et al., 2017b). Atlason et al. (2019) applied CNNs for tissue and white matter hyperintensity (WMH) segmentation in brain MRI scans. A Euclidean distance-based clustering method has also been employed to detect MS lesions in MRI images (Cetin et al., 2020). An unsupervised approach has been developed to quantitatively assess MS lesion progression to extract brain tissue distortions from MRI scans (Rachmadi et al., 2020). Furthermore, Seg-JDOT, a domain adaptation-based MS lesion segmentation framework, has demonstrated promising results (Ackaouy et al., 2020). However, unsupervised models generally exhibit lower accuracy and often require human intervention to align with domain-specific knowledge.

DL has significantly advanced the extraction of MS lesions by enabling automated feature learning directly from raw MRI scans. Convolutional neural networks (CNNs), especially the UNet architecture and its derivatives, have demonstrated exceptional performance in segmenting MS lesions (Ronneberger et al., 2015; Isensee et al., 2018). Attention-UNet enhances segmentation precision by focusing on lesion regions (Aslani et al., 2019), while DenseUNet and ResUNet improve feature propagation and gradient flow (Oktay et al., 2018; Jha et al., 2019; Long et al., 2015). Fully Convolutional Networks (FCNs) and encoder-decoder architectures have further refined lesion identification (Milletari et al., 2016; Chen et al., 2017). Furthermore, hybrid models incorporating Atrous Spatial Pyramid Pooling (ASPP) and Vision Transformers (ViTs) have demonstrated superior lesion detection capabilities by capturing long-range dependencies and multi-scale contextual information (He et al., 2023; Dosovitskiy et al., 2021; Azad et al., 2024). A hybrid architecture combining the SWin transformer and UNet models is effectively used for the identification of MS lesions (Nouman et al., 2024). Similarly, Davarani et al. (2025) have designed a hybrid architecture of transformers and autoencoders to segment MS lesions. A detailed comparative analysis of various techniques for identifying MS lesions from brain MRI images is presented in Table 1. The performance of all methods presented in Table 1 is evaluated using the Dice-score metric, which eliminates the issue of class imbalance in MS lesion datasets. Despite their success, DL models face challenges such as data scarcity, high computational costs, and overfitting on small training datasets.

**Table 1:** Overview of some of the well-known techniques of MS lesion segmentation from brain MRI images.

**References**	**Dataset**	**Method**	**Limitations**	**Dice score**
Isensee et al. (2018)	Local clinical dataset of 200 patients	nnUNet	Unable to detect small lesions	0.710
He et al. (2023)	Local clinical dataset of 150 patients	Vision Transformers based technique	High computational complexity and requires a large amount of data, suspected of overfitting	0.723
Azad et al. (2024)	ISBI	Vision transformers based framework	High computational cost and suspected of overfitting, require high volume of data	0.756
Nouman et al. (2024)	Local clinical dataset of 100 patients	Hybrid framework of UNet and Swin Transformer	Limited generalizability	0.748
Aslani et al. (2019)	ISBI dataset	Attention based UNet	Limited generalizability	0.694
Davarani et al. (2025)	Private dataset of 174 MS patients	TransUNet—a transformer-based encoder	Computational overhead and requires large data	0.712
Birenbaum and Greenspan (2016)	ISBI 2015	CNN	Limited generalization	0.6271
Valverde et al. (2017a)	ISBI 2015, MICCAI 2008	Cascaded 3D CNN	Modality dependence	0.6294
Ansari et al. (2021)	ISBI 2015	Inception module with CNN	Limited validation across datasets	0.6306
Beaumont and Greenspan (2016)	MICCAI 2016	CNN	Limited generalization	0.5623
Vera-Olmos et al. (2016)	MICCAI 2016	Random forest	Modality dependence	0.5537
Mahbod et al. (2016)	MICCAI 2016	ANN	Limited applicability and unable to capture fine details	0.5742
Kaur et al. (2024)	MICCAI 2008 and 2016	Patch-wise deep neural network	Unable to capture the fine lesion details and dataset-specific	0.870
McKinley et al. (2021)	MICCAI 2021	Deep CNN	Computational complexity	0.638
Basaran et al. (2022)	MICCAI 2021	Augmentation-based Deep CNN	Limited generalizability	0.510

Given the limitations of traditional supervised and unsupervised methods, recent research has focused on designing advanced DL designs to improve MS lesion segmentation. To address the limitations of the existing MS lesion segmentation methods discussed above, an efficient architecture, MS-DASPNet, is designed to extract lesions from brain MRI images by fusing low-level and high-level features through a dual-headed attention mechanism, which incrementally weights each channel to enhance feature representation. The encoder of the proposed model is initialized with VGG-16, which provides a strong hierarchical feature extraction capability due to its deep convolutional layers and pre-trained weights, enabling better generalization and improved feature reuse. Additionally, an ASPP section is integrated into the bottleneck to efficiently model contextual information across multiple spatial scales and improve lesion delineation across different resolutions. By addressing the weight update problem, the network further enhances its feature extraction efficiency. MS-DASPNet is computationally efficient, requiring fewer parameters, while the combination of VGG-16's hierarchical feature learning and ASPP's multi-scale context aggregation leads to substantial improvements in MS lesion delineation performance. The major contributions of the proposed architecture are as follows:

1. In this study, a DL based model, MS-DASPNet, is designed to segment MS lesions from brain MRI images. The proposed method is computationally efficient and enhances multi-scale contextual understanding using dilated convolutions, improving localization and global perception while mitigating spatial information loss.
2. The introduction of a dual-headed attention block in the skip connections of MS-DASPNet enhances feature refinement by enabling the model to focus on both spatial and channel-wise dependencies, leading to improved accuracy and boundary precision.
3. To assess the generalizability and robustness of MS-DASPNet, experiments are conducted on four publicly available datasets (ISBI-2015, Mendeley, MICCAI-2016, and MICCAI-2021), and performance is evaluated using quantitative metrics.
4. The performance of the MS-DASPNet is matched with the existing DL architectures, including UNet, Attention UNet, DenseUNet, and Res-UNet, for MS lesion extraction in brain MRI scans.

The remainder of the paper is structured as follows: The Section 2 outlines the four datasets utilized in this study. The Section 3 describes the architectural design and methodological framework of the proposed approach. The Section 4 details the experimental setup and presents the quantitative and qualitative segmentation outcomes. The Section 5 provides an in-depth evaluation of the proposed model against various state-of-the-art DL frameworks. Finally, the Section 6 summarizes the key findings and outlines potential directions for future work.

## 2 Dataset description

This section outlines the datasets used for the segmentation of MS lesions from brain MRI scans as presented in Table 2.

**Table 2:** Summary of datasets used in the study.

**Dataset**	**Modalities**	**No. of cases**	**MRI scanner**
ISBI-2015	T2-w, MPRAGE, PD, FLAIR	20	3T Philips
Mendeley	T1-w, T2-w, FLAIR	60	1.5 Tesla
MICCAI-2016	T1-w, T2-w, FLAIR, DP, GD	40	Siemens Verio 3T
MICCAI-2021	FLAIR	40	1.5T and 3T GE, Philips, Siemens

### 2.1 ISBI-2015 dataset

**Figure 1:**
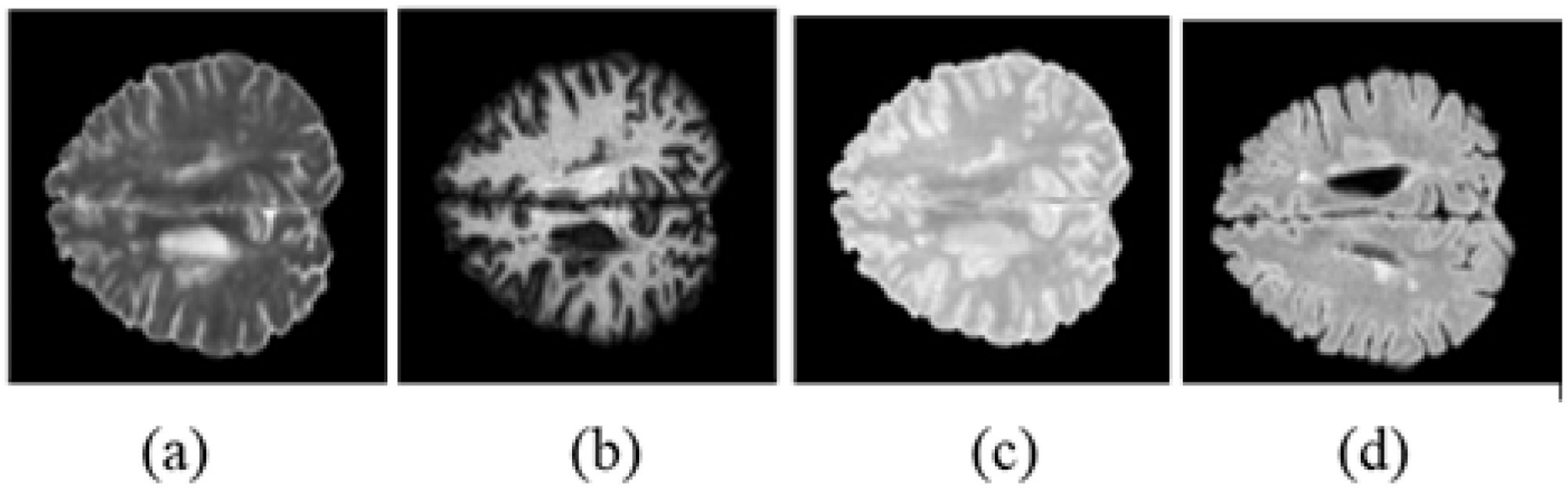
ISBI-2015 sample brain MRI images in different image modalities when slice number is 100 for the first patient. **(a)** T2-weighted, **(b)** MPRAGE, **(c)** PD, **(d)** FLAIR.

In 2015, the International Symposium on Biomedical Imaging hosted the Longitudinal MS Lesion Segmentation Challenge, providing training and test data for brain MRI images acquired using a 3T Philips MRI scanner (Carass et al., 2017). The dataset includes 3D (NIfTI) brain MRI images from five patients, acquired at four time points across multiple modalities, including T2-weighted, FLAIR, MPRAGE, and proton-density-weighted scans. Although MS lesions are present in all modalities, the FLAIR modality has been chosen for MS lesion segmentation due to its clear visibility, as illustrated in Figure 1.

### 2.2 Mendeley dataset

**Figure 2:**
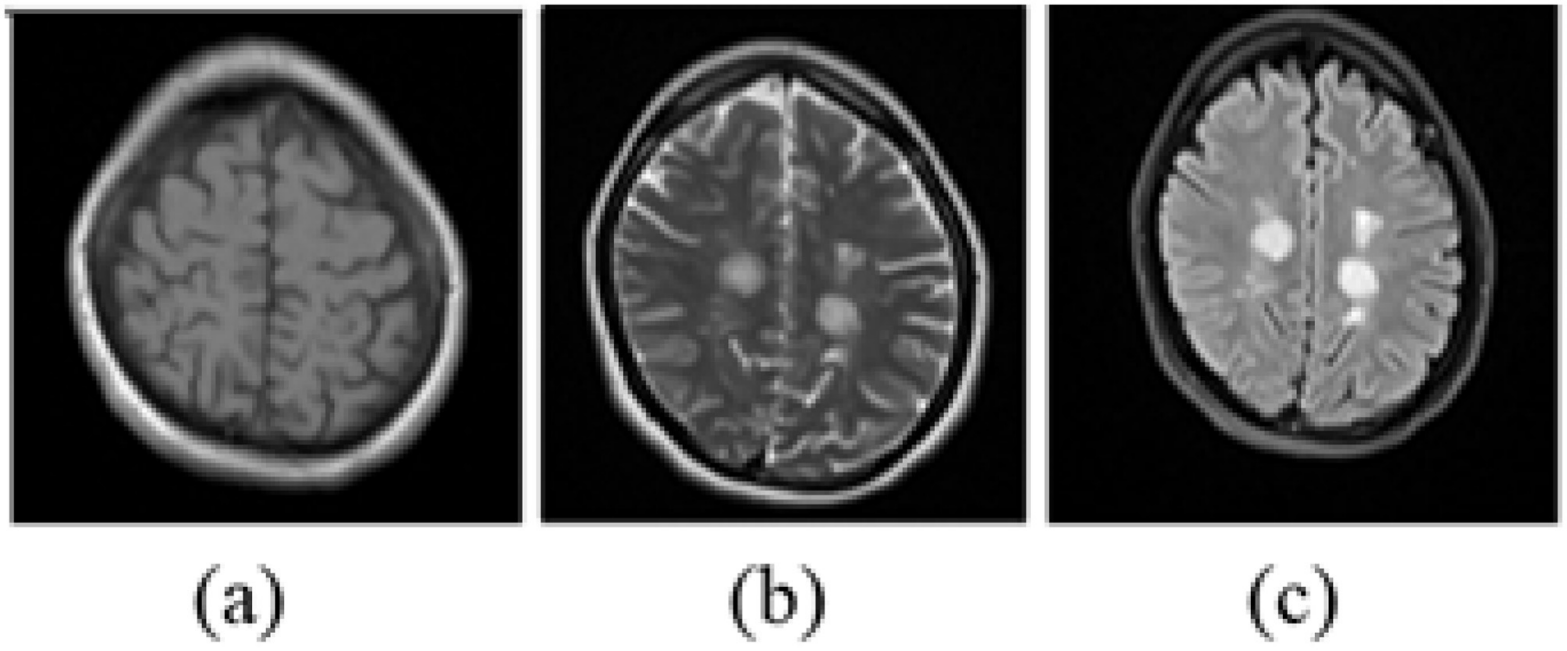
Mendeley dataset sample brain MRI in different image modalities when slice number is 15 for the second patient in the dataset. **(a)** T1-weighted, **(b)** T2-weighted. **(c)** FLAIR.

The Brain MRI Dataset of MS with Consensus Manual Lesion Segmentation and Patient Meta Information (Muslim et al., 2022) is employed for extracting MS lesions from MRI scans. This dataset includes 3D brain MRI volumes in NIfTI format from 60 patients, acquired using multiple imaging modalities, including T1-weighted, T2-weighted, and FLAIR. Each 3D volume represents a unique patient and varies in spatial dimensions. Although MS lesions are observable across all three modalities, the FLAIR modality is selected for segmentation due to its superior lesion visibility, as demonstrated in Figure 2.

### 2.3 MICCAI-2016/MSSEG-2016

This dataset includes three-dimensional (NIfTI) MRI scans in various modalities, including:

1. 3D FLAIR Image
2. 3D T1 Image
3. T2 Image
4. DP Image
5. 3D T1 Gd Image (Post-contrast agent image).

The training set includes 15 images, and the testing set includes 34 images, with their corresponding true labels. After slicing each 3D image, we generated a total of 2,432 images and their corresponding masks. The true lesion masks were provided by seven experts, and a consensus mask was used to evaluate the results. We used preprocessed 3D images for slicing in the FLAIR modality.

**Figure 3:**
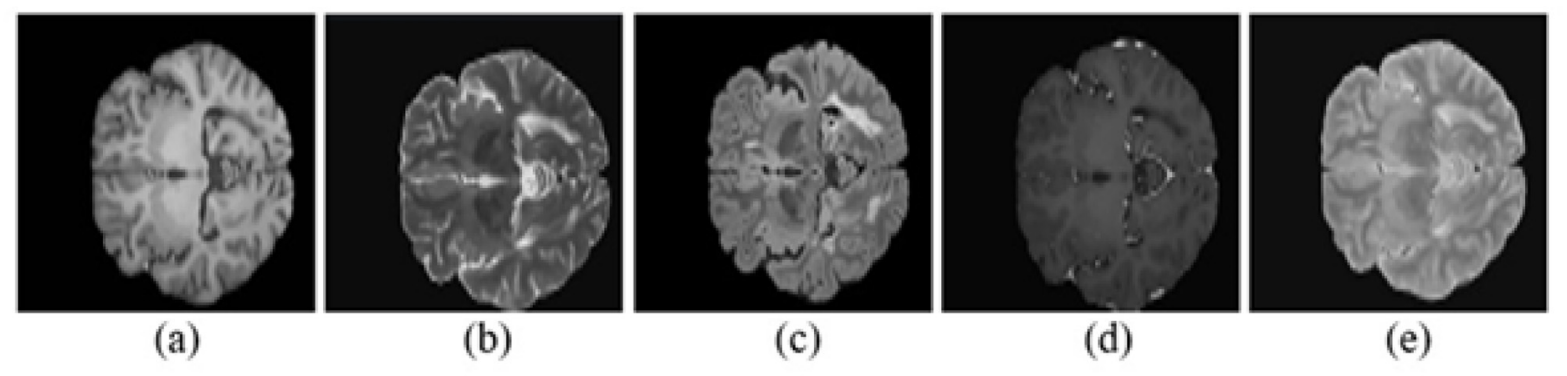
MICCAI-2016 dataset sample brain MRI in different image modalities when slice number is 282 for the third patient in the dataset. **(a)** T1-weighted. **(b)** T2-weighted. **(c)** FLAIR. **(d)** T1-Gd (gadolinium contrast). **(e)** DP-proton density.

The images of the MSSEG-2016 dataset (Commowick et al., 2021a) are shown in Figure 3.

### 2.4 MICCAI-2021/MSSEG-2

**Figure 4:**
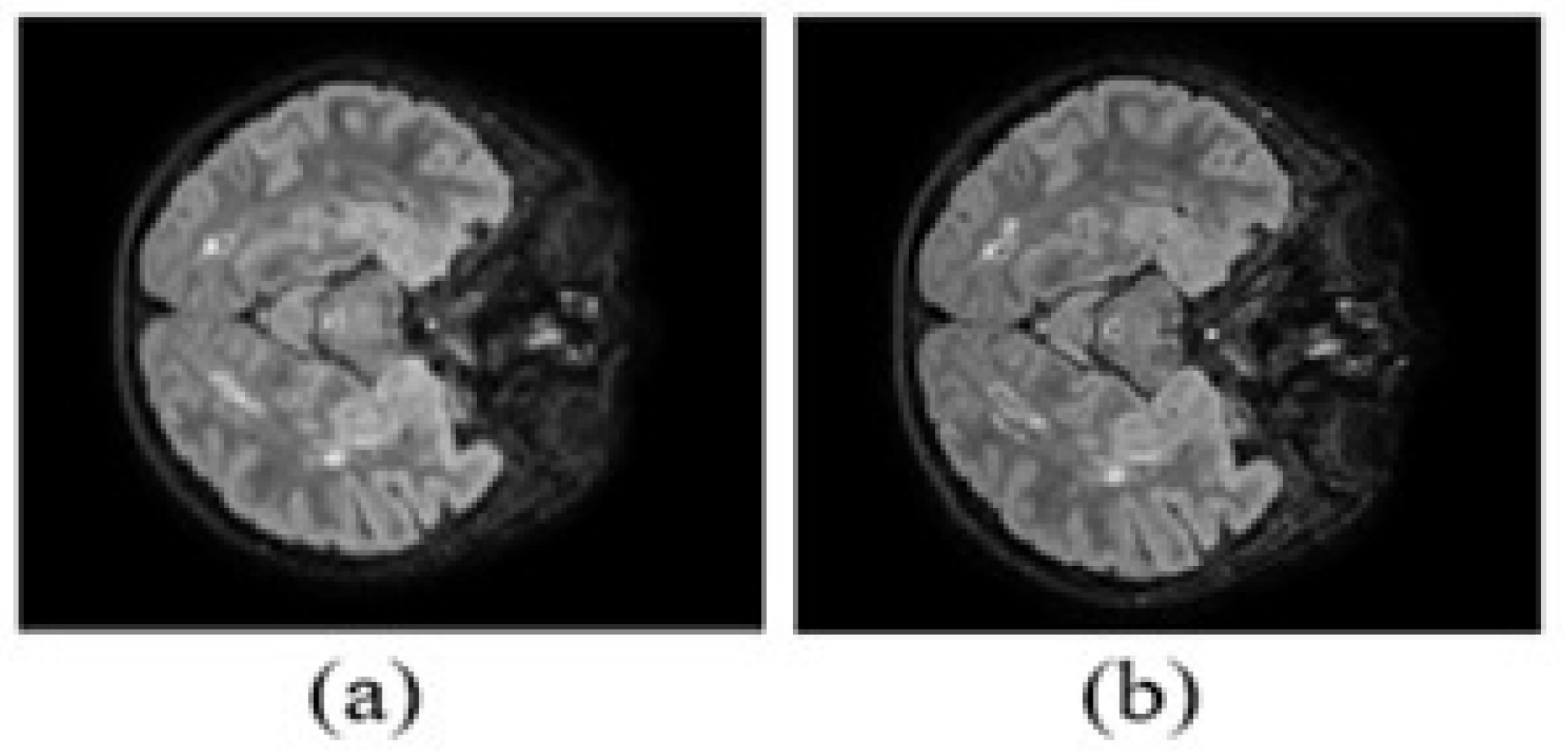
MICCAI-2021 dataset sample brain MRI in FLAIR modalities when slice number is 241 for the thirty-fifth patient in the dataset. **(a)** Time point-1 image. **(b)** Time point-2 image.

This dataset comprises MR neuroimaging data from 40 patients, each having undergone 3D FLAIR acquisitions at two distinct time points, with variable intervals between scans. MS lesions were segmented by four experts, and a majority vote was applied voxel-by-voxel to generate the final consensus masks, which serve as the basis for MS lesion segmentation. This study used only second-time-point images for lesion segmentation. The dataset, referred to as MSSEG-2, is publicly available at Commowick et al. (2021b). Representative images of the data set are shown in Figure 4.

## 3 Proposed method

**Figure 5:**
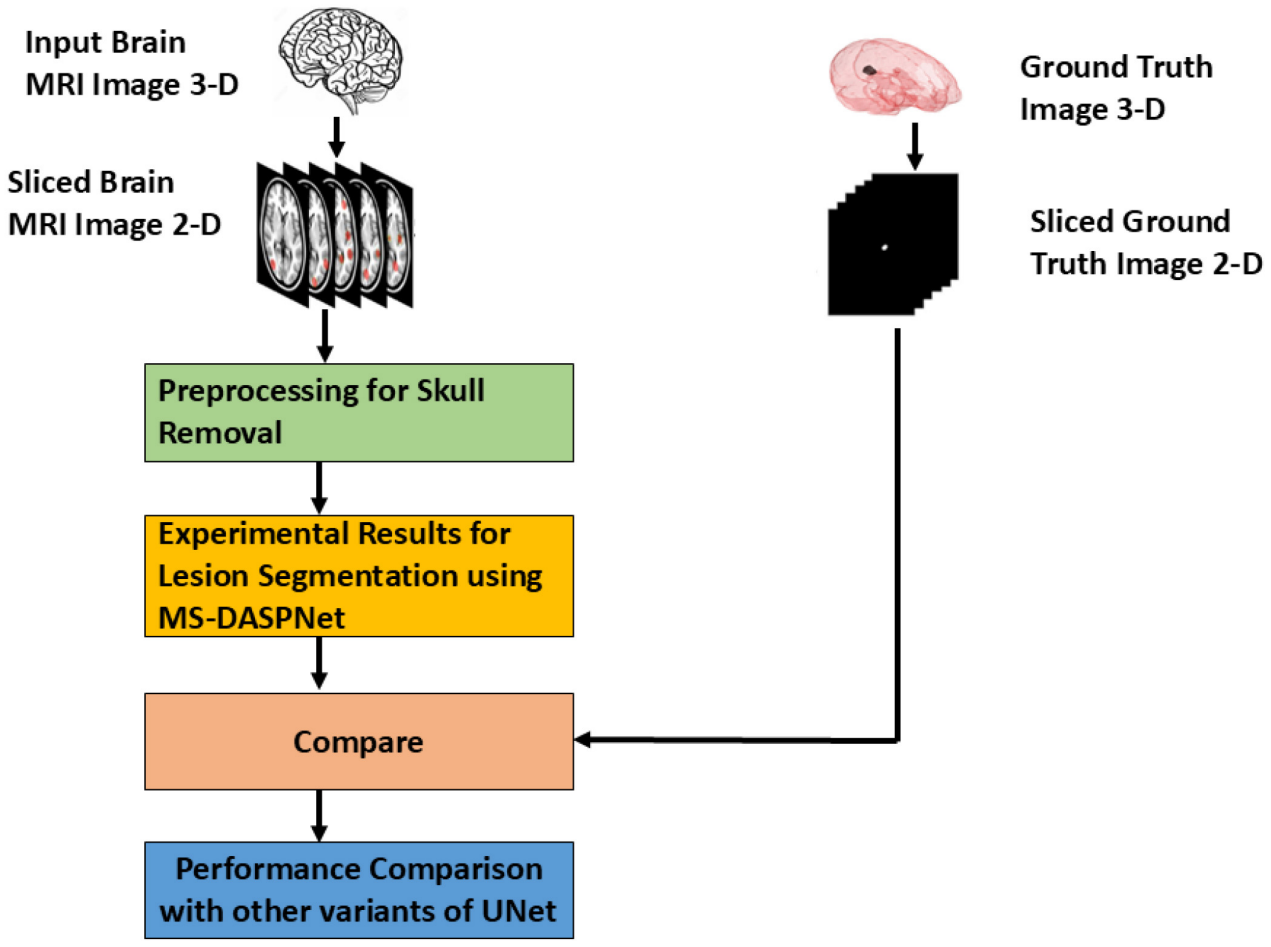
Workflow of MS-DASPNet approach.

**Figure 6:**
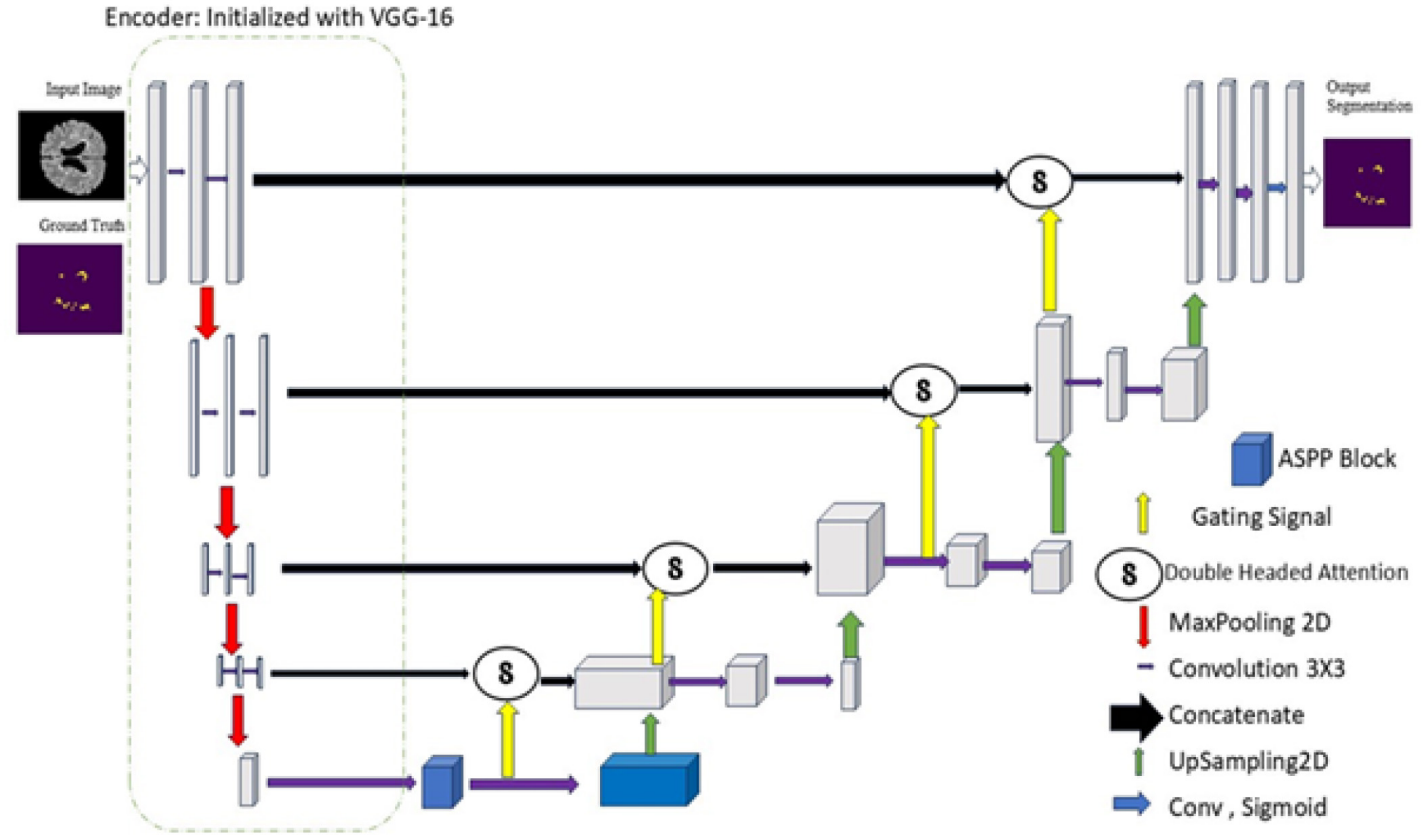
Proposed model for MS lesion segmentation (encoder initialized with VGG- 16).

This work proposes and evaluates MS-DASPNet, a novel deep-learning framework, on four distinct datasets: ISBI-2015, Mendeley Dataset, MICCAI-2016, and MICCAI-2021. For analysis, pixel-based classification uses DL techniques such as UNet, DenseUNet, Attention UNet, ResUNet, and MS-DASPNet. Firstly, three-dimensional brain MRI scans have been sliced to produce two-dimensional MRI images. Preprocessing includes a sequence of steps for skull removal on the dataset: (i) contrast elongation, (ii) histogram equalization, (iii) Otsu thresholding, and (iv) morphological operations. The flowchart depicting the sequence of steps involved in evaluating the proposed architecture is shown in Figure 5. The architecture of the MS-DASPNet model is presented in Figure 6 and described in Algorithm 1, and the notations used are presented in Table 3.

Algorithm 1 MS-DASPNet: dual attention and spatial pyramid pooling for MS lesion segmentation.

**Input**: 3D brain MRI image *I*_3*D*_, ground truth mask 
           *M*_3*D*_, VGG-16 parameters Θ_*v*_, dilation rates *d* **Output**: Predicted segmentation mask Ŝ 
**1. Preprocessing:** 
   (a) Convert *I*_3*D*_ to 2D slices {*I*_*i*_} 
   (b) Apply NL-means denoising 
   (c) Apply N4 bias field correction 
   (d) Resize *I*_*i*_ and *M*_*i*_ to 256 × 256 
**2. Feature extraction:** 
   (a) Load pre-trained VGG-16 (without FC layers) 
   (b) Extract encoder features *E*_1_, *E*_2_, *E*_3_, *E*_4_ using Θ_*v*_ 
**3. ASPP bottleneck:** 
    (a) Apply atrous convolutions with *r* ∈ {1, 6, 12, 18} 
   (b) Concatenate resulting feature maps 
   (c) Apply 1 × 1 convolution to obtain *B*_*f*_ **4. Decoder with dual attention:** 
   (a) Upsample decoder feature *D*_*k*+1_ to obtain *U*_*k*_ 
   (b) Generate channel attention map *M*_*c*_ 
   (c) Generate spatial attention map *M*_*s*_
   (d) Compute attention-weighted feature *A*_*k*_ = *M*_*c*_·*M*_*s*_·*U*_*k*_ 
   (e) Fuse with corresponding encoder feature: *D*_*k*_ = Concat(*A*_*k*_, *E*_*k*_)
**5. Output:** 
   (a) Apply 1 × 1 convolution on *D*_4_ 
   (b) Apply sigmoid activation to obtain Ŝ 
**6. Evaluation:**
   Compute Dice, Jaccard, Precision, Sensitivity, and Specificity

**Table 3:** Notations and their descriptions used in the MS-DASPNet algorithm.

**S.No**	**Notation**	**Description**
1	*I*_3*D*_	Input 3D brain MRI image
2	*M*_3*D*_	Ground truth lesion mask for the 3D MRI image
3	*I*_*i*_	2D axial slice extracted from *I*_3*D*_
4	*M*_*i*_	2D ground truth mask corresponding to *I*_*i*_
5	Θ_*v*_	Parameters of the pre-trained VGG-16 model
6	*E*_1_, *E*_2_, *E*_3_, *E*_4_	Encoder features extracted from different VGG-16 blocks
7	*B*_*f*_	Bottleneck feature map after ASPP and 1 × 1 convolution
8	*U*_*k*_	Upsampled decoder feature map at level *k*
9	*M*_*c*_	Channel attention map generated from *U*_*k*_
10	*M*_*s*_	Spatial attention map generated from *U*_*k*_
11	*A*_*k*_	Attention-weighted decoder feature map at level *k*
12	*D*_*k*_	Decoder feature map after attention and skip connection at level *k*
13	Ŝ	Final predicted segmentation mask

### 3.1 Preprocessing

In this step, the three-dimensional brain MRI volumes were sliced to obtain a two-dimensional representation suitable for model training. Each MRI volume and its corresponding ground-truth mask consist of approximately 300–400 slices in the FLAIR sequence. Since the proposed MS-DASPNet operates on 2D inputs, the 3D brain MRI volumes were first decomposed into axial slices, as the axial plane provides superior visualization of MS lesions. To ensure that the selected slices contained meaningful information, only slices exhibiting MS lesions with a minimum lesion size of five pixels in the corresponding ground-truth masks were retained. Furthermore, instead of using all slices from each volume, approximately 65%–70% of the axial slices were utilized for the experiments, as the initial and terminal slices predominantly correspond to peripheral brain regions and generally do not contain visible lesions. This strategy enabled the selection of slices that contained the maximum lesion information. The sliced brain MRI images obtained are affected by noise and require skull removal and extraction of brain tissues by applying the preprocessing. This is achieved by denoising each image using the NL-means algorithm (Coupé et al., 2008), and the volBrain platform (Manjón and Coupé, 2015) is used to extract the brain from the image. Finally, the bias correction is performed using the N4 algorithm (Tustison et al., 2010). The 2D images of all datasets, along with their masks, are resized to 256 × 256.

### 3.2 Data augmentation

Generally, deep learning models suffer from issues of overfitting due to the small size of the dataset. This issue can be resolved by data augmentation, which will improve the model's generalization ability and increase its robustness to handle complex features. In this work, the following operations are performed to expand the dataset size.

1. Rotation—Rotation has been performed at the angles of 45, 90, and 125 degrees on both the brain MRI image and the mask image.
2. Scaling—Scaling has been performed with a scale factor of 1.5 and 2 on both the images of brain MRI as well as the mask image.
3. Translation—Translation has been performed to translate an image horizontally on both images.

### 3.3 Overview of the MS-DASPNet architecture

**Figure 7:**
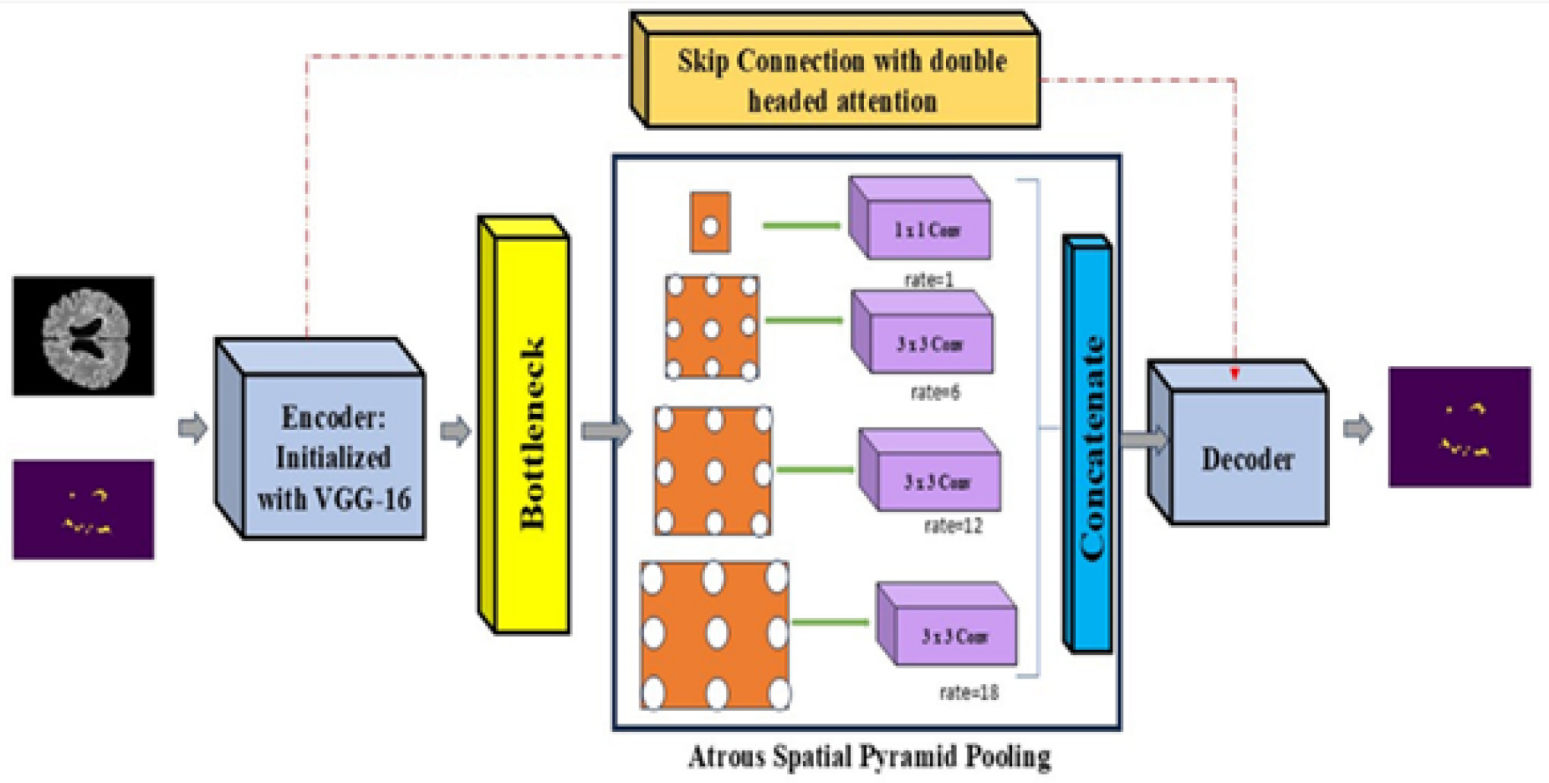
MS-DASPNet architecture.

This study proposes an MS-DASPNet structure—A Transfer Learning-based UNet model incorporating ASPP and Dual-Headed Attention, an effective DL design for MS lesion identification in brain MRI. The proposed model builds upon the standard UNet by integrating dual-headed attention mechanisms in skip connections and utilizing a pre-trained VGG-16 encoder for feature extraction. Additionally, ASPP is embedded in the bottleneck layer to capture multi-scale contextual information from the deepest encoder feature map. These enhancements address key limitations of conventional UNet models, including feature loss and poor boundary depiction, which are critical in medical image segmentation tasks. The architectural modifications introduced in our model aim to improve feature representation, spatial attention, and multi-scale feature learning, thereby enhancing segmentation accuracy. Figure 7 illustrates the overall network architecture, which consists of:

1. An encoder initialized with VGG-16, employing pre-trained feature representations for robust hierarchical feature extraction.
2. Dual-headed attention modules integrated within skip connections to enhance feature fusion and suppress irrelevant activations.
3. An Atrous Spatial Pyramid Pooling (ASPP) component embedded in the bottleneck to extract multi-scale contextual features from deep encoder representations.

By incorporating these improvements, the proposed model outperforms the UNet model variants, especially in detecting complex lesion boundaries.

In the preprocessing stage, each 3D MRI volume (*I*_3*D*_) is converted into 2D slices, denoised using NL-means filtering, corrected for intensity inhomogeneity via N4 bias field correction, and resized to 256 × 256. Encoder features (*E*_1_–*E*_4_) are extracted from a pre-trained VGG-16 network. The ASPP module aggregates multi-scale context using dilation rates 1, 6, 12, 18, followed by 1 × 1 convolution for feature refinement. In the decoder, both channel and spatial attention mechanisms are applied to enhance lesion-relevant regions before skip connections are fused. The final segmentation mask (Ŝ) is produced using a 1 × 1 convolution followed by a sigmoid activation.

### 3.4 Network architecture

The proposed model follows a U-shaped network architecture, where the encoder is initialized with VGG-16, the ASPP module is integrated into the bottleneck, and double-headed attention is applied to each skip connection to enhance feature extraction and preserve critical details.

#### 3.4.1 Encoder with VGG-16

The encoder in our proposed Transfer Learning-based UNet is initialized with VGG-16, a deep CNN originally designed for image classification, proposed by the Visual Geometry Group (VGG). VGG-16 is defined by its depth, comprising 16 layers, with 13 convolutional layers and three fully connected layers. Instead of training the encoder from scratch, the proposed model used pre-trained weights from the ImageNet dataset, enabling it to efficiently capture hierarchical feature representations. VGG-16 is chosen as the feature extraction network due to its strong ability to capture rich hierarchical features through its deep, pretrained architecture. It provides robust and transferable representations that generalize well across diverse medical imaging modalities, making it highly effective for image segmentation tasks. The VGG-based encoder extracts multi-scale features at different depths, which are later utilized in the UNet decoder through skip connections.

The encoder follows the architecture of VGG-16, which consists of five convolutional blocks, each containing multiple convolutional layers followed by ReLU activation and max pooling for downsampling. Let *I*∈ℝ^*H*×*W*×*C*^ be the input, where *H* is the height, *W* is the width, and *C* is the number of channels of the input MRI image. The feature extraction process through a convolutional layer is described in Equation 1:


$$\begin{array}{l} F_{l}=\sigma \left(W_{l}*F_{l-1}+b_{l}\right) \end{array}$$ (1)

where:

- *F*_*l*_ represents the feature map at layer *l*,
- *W*_*l*_ and *b*_*l*_ are the pretrained weights and biases,
* denotes the convolution operation,
- σ is the ReLU activation function, defined as σ(*x*) = max(0, *x*).

The max pooling operation reduces the spatial dimensions by a factor of 2 at each block, as shown in Equation 2:


$$\begin{array}{l} F_{l}^{\prime }=\max\left(F_{l}\left(i,j\right)\right)where:F_{l}^{\prime }isthepooledfeaturemap \end{array}$$ (2)

**Figure 8:**
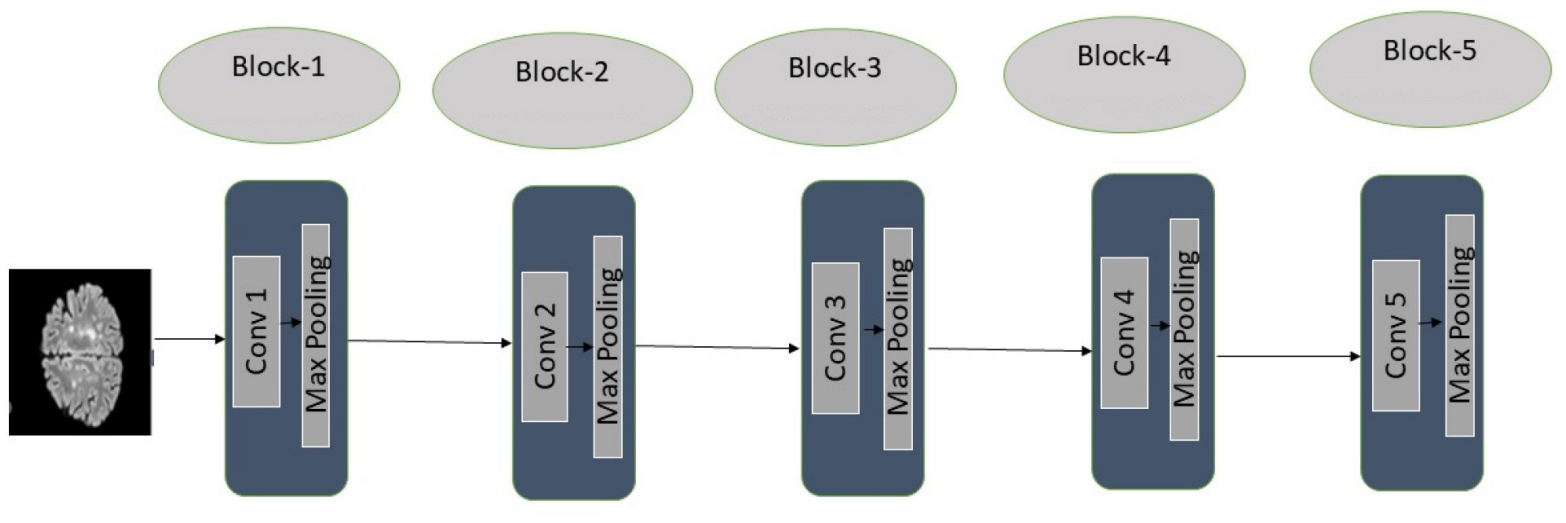
Feature extraction using VGG-16.

Since VGG-16 was pre-trained on ImageNet, we freeze its layers during the initial training to retain the learnt feature representations while preventing unnecessary weight updates. Using VGG-16 as the encoder effectively captures both low-level and high-level features, as the model benefits from being pre-trained on the large and diverse ImageNet dataset. The complete feature extraction process through VGG-16 on a sample brain MRI image has been demonstrated in Figure 8.

#### 3.4.2 Atrous Spatial Pyramid Pooling (ASPP)

ASPP is an advanced feature extraction method that captures multi-scale contextual information by employing multiple dilated convolutions with varying dilation rates. Integrating ASPP into the bottleneck of the architecture enhances the model's capacity to aggregate features across multiple receptive field scales while maintaining computational efficiency. The bottleneck layer represents the deepest feature-extraction stage, where spatial resolution is substantially reduced, resulting in the loss of fine-grained details and contextual information. Incorporating ASPP into the bottleneck addresses these challenges by (i) expanding the receptive field without increasing the parameter count, (ii) capturing both local and global contextual features, and (iii) retaining fine details essential for accurate lesion extraction.

ASPP applies parallel atrous convolutions with different dilation rates to the same feature map, enabling multi-scale feature extraction. Let *F*_ASPP_ denote the output feature map after applying ASPP, *F*_bottleneck_ be the input feature map from the VGG-16 encoder, and *W*_*i*_, *b*_*i*_ be the weights and biases of the atrous convolution filters at different scales. The convolution with an atrous rate *r*_*i*_ is denoted as *_r_*i*__. The number of atrous convolutions with different dilation rates is represented by n. The feature map computed by ASPP is represented in Equation 3:


$$\begin{array}{l} F_{\text{ASPP}}=\overset{n}{\underset{i=1}{\sum }}\left(W_{i}{*}_{r_{i}}F_{\text{bottleneck}}+b_{i}\right) \end{array}$$ (3)

ASPP includes a 1 × 1 convolution (*r* = 1) for local feature extraction. Three atrous convolutions with dilation rates of *r* = 6, 12, and 18 for multi-scale context. Global average pooling to incorporate global image context. Concatenation of all resulting feature maps followed by a 1 × 1 convolution to fuse the information. The final output of the ASPP module is computed as in Equation 4:


$$\begin{array}{l} F_{\text{out}}=\sigma \left(W_{c}\cdot \left[F_{1},F_{6},F_{12},F_{18},F_{\text{GAP}}\right]+b_{c}\right) \end{array}$$ (4)

Where *F*_1_, *F*_6_, *F*_12_, and *F*_18_ denote the outputs of convolutions with dilation rates of 1, 6, 12, and 18, respectively, *F*_GAP_ represents the global average pooled feature map, [·] indicates channel-wise concatenation, *W*_*c*_ and *b*_*c*_ are the learnable weights and biases for feature fusion, and σ denotes the ReLU activation function.

ASPP enhances multi-scale feature learning across different spatial resolutions, improves boundary distinction, and maintains computational efficiency because it does not significantly increase the number of model parameters.

#### 3.4.3 Double-headed attention (DHA)

**Figure 9:**
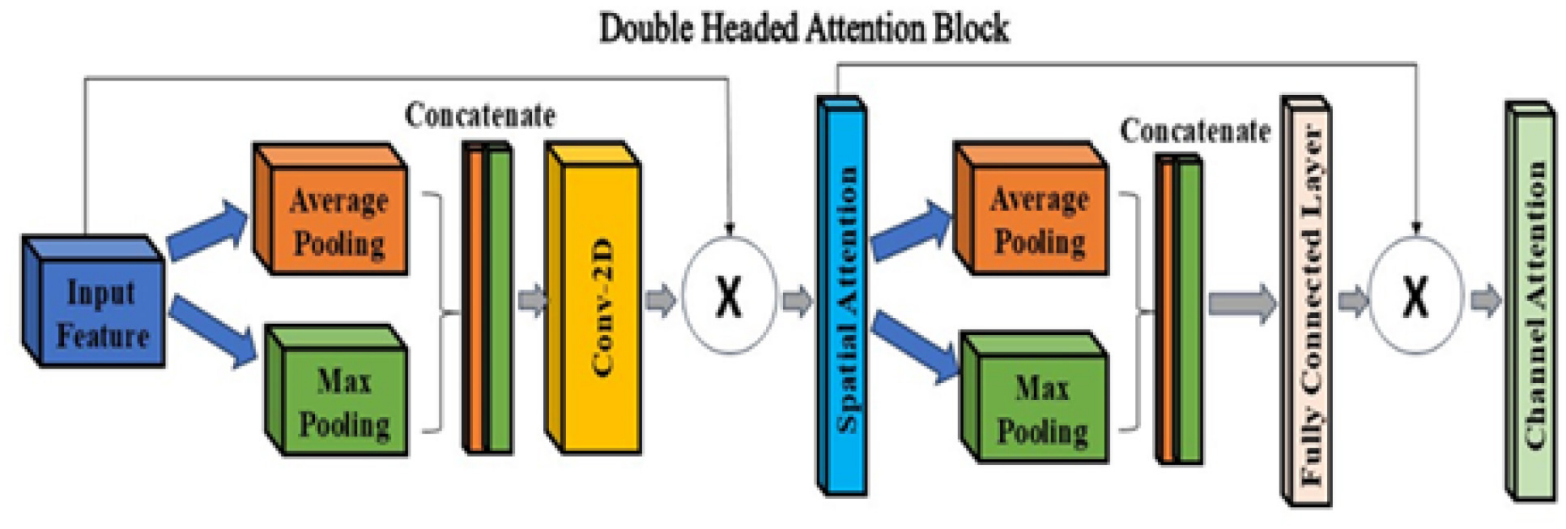
Double-headed attention block.

In the proposed architecture, the DHA block refines skip connections, effectively integrating channel and spatial attention for enhanced feature representation. Spatial attention highlights important regions in the feature map, whereas channel attention prioritizes the most relevant feature channels. This dual attention mechanism improves feature selection and preservation, improving lesion identification accuracy. The architecture of DHA is described in Figure 9.

The spatial attention mechanism highlights regions of interest by applying Global Average Pooling (GAP) and Global Max Pooling (GMP) across spatial dimensions. The resulting attention map is then used to refine the input feature map, emphasizing spatially significant areas.

Given an input feature map *X*∈ℝ^*H*×*W*×*C*^, spatial attention is computed as:


$$\begin{array}{l} F_{\text{avg}}=\frac{1}{C}\overset{C}{\underset{c=1}{\sum }}X_{h,w,c},\;F_{\text{max}}=\max\left(X_{h,w,c}\right) \end{array}$$ (5)

where *F*_avg_ and $F_{\text{max}}\in {\mathbb{R}}^{H\times W\times 1}$ are the global average pooled and global max pooled feature maps, respectively. These maps are concatenated and passed through a 7 × 7 convolution followed by a sigmoid activation to obtain the spatial attention mask *A*_*s*_, as defined in Equation 6:


$$\begin{array}{l} A_{s}=\sigma \left(f_{\text{conv}7\times 7}\left(\left[F_{\text{avg}},F_{\text{max}}\right]\right)\right) \end{array}$$ (6)

where σ denotes the sigmoid activation function. The final attention-weighted feature map *X*_*s*_ is obtained by:


$$\begin{array}{l} X_{s}=A_{s}⊙X \end{array}$$ (7)

where ⊙ represents element-wise multiplication. This mechanism enhances important spatial regions in the feature map, improving the model's focus on relevant areas during extraction.

The channel attention mechanism provides importance to each feature channel using Global Average Pooling (GAP) and Global Max Pooling (GMP) across spatial dimensions.

Given an input $X_{s}\in {\mathbb{R}}^{H\times W\times C}$ (from the spatial attention module), channel descriptors are computed as:


$$\begin{array}{l} F_{\text{avg}}^{c}=\frac{1}{H\times W}\overset{H}{\underset{h=1}{\sum }}\overset{W}{\underset{w=1}{\sum }}X_{h,w,c} \end{array}$$ (8)

$$\begin{array}{l} F_{\text{max}}^{c}=\underset{h,w}{\max}X_{h,w,c} \end{array}$$ (9)

where $F_{\text{avg}}^{c}$ and $F_{\text{max}}^{c}$ represent the globally averaged and maximized pooled channel features, respectively.

These descriptors are concatenated and passed through a two-layer dense network as follows:


$$\begin{array}{l} A_{c}=\sigma \left(W_{2}\cdot \text{ReLU}\left(W_{1}\left[F_{\text{avg}}^{c},F_{\text{max}}^{c}\right]\right)+b_{2}\right) \end{array}$$ (10)

where *W*_1_ and *W*_2_ are learnable weight matrices, *b*_2_ is the bias term, and σ denotes the sigmoid activation function. The final channel-wise attention-weighted feature map is obtained as:


$$\begin{array}{l} X_{c}=A_{c}⊙X_{s} \end{array}$$ (11)

where ⊙ denotes element-wise multiplication, applying the attention weights to refine the features along the channel dimension.

After Dual-Headed Attention (DHA) is applied to each skip connection, the enhanced feature maps are concatenated with the upsampled decoder features. This process is represented in Equation 12, where $F_{\text{skip}}^{i}$ denotes the DHA-enhanced skip connection feature map, and $F_{\text{dec}}^{i}$ is the upsampled decoder feature map at level *i*:


$$\begin{array}{l} F_{\text{merged}}^{i}=\text{Concatenate}\left(F_{\text{dec}}^{i},F_{\text{skip}}^{i}\right) \end{array}$$ (12)

This fusion of encoder and decoder features enables richer contextual representation and more precise localization, which is crucial for accurate detection.

### 3.5 Loss function

Binary Cross-Entropy (BCE) is a loss function and serves as the primary objective for training, quantifying the divergence between the predicted probability map and the actual ground-truth labels. Given a ground truth mask *y*_*i*_ and its corresponding predicted output *o*_*i*_, the BCE loss is mathematically expressed as follows:


$$\begin{array}{l} \text{BCE}=-\underset{i}{\sum }\left[y_{i}\log o_{i}+\left(1-y_{i}\right)\log\left(1-o_{i}\right)\right] \end{array}$$ (13)

BCE loss is particularly advantageous for tasks that require classifying each pixel as belonging to either the object of interest (foreground) or the background. Since binary segmentation is inherently a pixel-wise classification problem, BCE is well-suited for optimizing the model's predictive capability. It treats each pixel independently, ensuring the network learns to effectively differentiate between the two classes. By reducing BCE loss during training, the model enhances its accuracy, making it a widely used loss function for binary applications.

### 3.6 Evaluation metrics

To evaluate performance, different performance parameters have been utilized, as mentioned in Equations 14–17.

- True positive (*T*_*P*_): MS pixel identified as MS
- True negative (*T*_*N*_): non-MS pixel identified as non-MS
- False negative (*F*_*N*_): MS pixel identified as non-MS
- False positive (*F*_*P*_): non-MS pixel identified as MS

$$\begin{array}{l} \text{Precision/correctness}=\frac{T_{P}}{T_{P}+F_{P}} \end{array}$$ (14)

$$\begin{array}{l} \text{Sensitivity/completeness}=\frac{T_{P}}{T_{P}+F_{N}} \end{array}$$ (15)

$$\begin{array}{l} \text{Quality/Jaccard}=\frac{T_{P}}{T_{P}+F_{N}+F_{P}} \end{array}$$ (16)

$$\begin{array}{l} \text{Dice score}=\frac{2\cdot T_{P}}{2\cdot T_{P}+F_{P}+F_{N}} \end{array}$$ (17)

## 4 Experimental results and discussion

This section outlines the experimental settings employed to simulate MS-DASPNet architectures, followed by a comprehensive analysis and discussion of the resulting experimental outcomes.

### 4.1 Experimental settings

The experiments are conducted on a GPU-equipped system featuring an Intel Core i5 processor at 2.5 GHz clock speed, 16 GB RAM, and an NVIDIA RTX graphics card. The details about the hyperparameters used for the training of the proposed model are presented in Table 4. All datasets are divided into training and testing subsets in a 90:10 ratio to ensure that no patient data overlaps between these sets to avoid data leakage. To prevent overfitting, a dropout rate of 0.2 is incorporated as a regularization technique, while a learning rate of 0.001 is used to ensure stable and efficient convergence. The model used convolutional filters of progressively increasing sizes (16, 32, 64, 128, and 256) to capture multi-scale features, along with a kernel size of 7 for enhanced feature extraction. A batch size of 4 was used, and the BCE loss was effectively selected for the lesion extraction task. The Adam optimizer, with an adaptive learning rate, is used over 100 epochs to fine-tune the model parameters, resulting in robust training and precise outcomes. The details regarding the number of subjects used for conducting experiments on each dataset are provided in Table 5.

**Table 4:** Hyperparameters used by MS-DASPNet.

**Hyperparameter**	**Value**
Loss function	Binary cross-entropy loss
Batch size	4
Optimizer	Adam
Learning rate	0.001
Dropout	0.2
Epochs	100

**Table 5:** Summary of the datasets used in this study.

**Subjects**	**ISBI-2015 dataset**	**Mendeley dataset**	**MICCAI-2016**	**MICCAI-2021**
Training subjects	108	266	1236	42
Testing subjects	12	30	138	5
Total subjects	120	296	1374	47

### 4.2 Experimental results and analysis

The proposed experiments are conducted on four datasets, namely ISBI-2015, Mendeley, MICCAI-2016, and MICCAI-2021.

#### 4.2.1 On ISBI-2015 dataset

**Figure 10:**
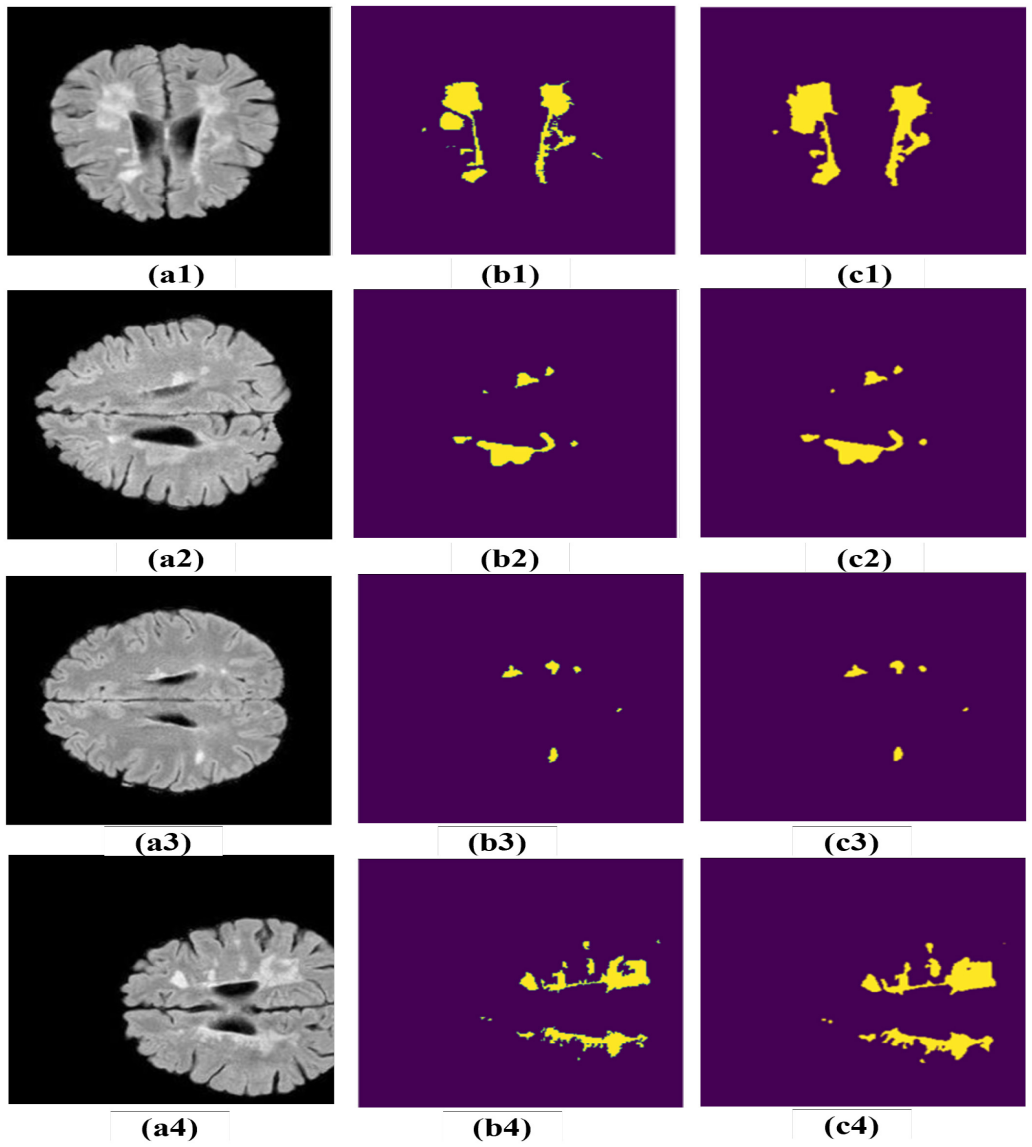
Results obtained on ISBI-2015 dataset. **(a1–a4)** Brain MRI image. **(b1–b4)** Corresponding ground truth image. **(c1–c4)** Segmentation obtained using MS-DASPNet.

The segmentation results produced by MS-DASPNet on the ISBI-2015 dataset are illustrated in Figure 10. Specifically, Figures 10a1–a4 displays four sample brain MRI images from the dataset, while Figures 10b1–b4 presents the corresponding ground truth masks used for performance evaluation. Figures 10c1–c4 shows the segmentation outputs generated by the MS-DASPNet architecture. Based on visual inspection of the MS lesions extracted by the proposed method, MS-DASPNet effectively identifies them. The performance is further evaluated using various quantitative parameters, and results are shown in Table 6. The experimental results reveal that the MS-DASPNet model demonstrates robust performance in MS lesion segmentation on the ISBI-2015 dataset, obtaining a Dice Score of 0.8329 and a Jaccard index of 0.733, which reflect strong spatial overlap between the predicted and reference lesion. The precision of 0.9252 indicates high confidence in positive lesion predictions, with minimal over-segmentation. A sensitivity of 0.7804 suggests effective lesion detection, capturing a majority of true lesion voxels, although with some under-segmentation in more diffuse or low-contrast regions. The specificity of 0.9987 and a false positive rate (FPR) of just 0.0013 confirm that the model maintains strong background suppression, crucial for minimizing false detections. These results highlight MS-DASPNet's ability to balance lesion sensitivity and anatomical specificity, making it well-suited for automated quantification of MS lesion load in clinical MRI scans.

**Table 6:** Experimental results of MS-DASPNet on different quantitative parameters.

**Dataset\parameter**	**Dice**	**Jaccard**	**Precision**	**Specificity**	**Sensitivity**	**FPR**
Mendeley	0.5076	0.3519	0.7278	0.9985	0.4357	0.0015
ISBI 2015	0.8329	0.7330	0.9252	0.9987	0.7804	0.0013
MICCAI 2016	0.8736	0.7982	0.8746	0.9995	0.8834	0.0005
MICCAI 2021	0.8706	0.7719	0.8943	0.9996	0.8503	0.0004

#### 4.2.2 On MICCAI-2016 dataset

**Figure 11:**
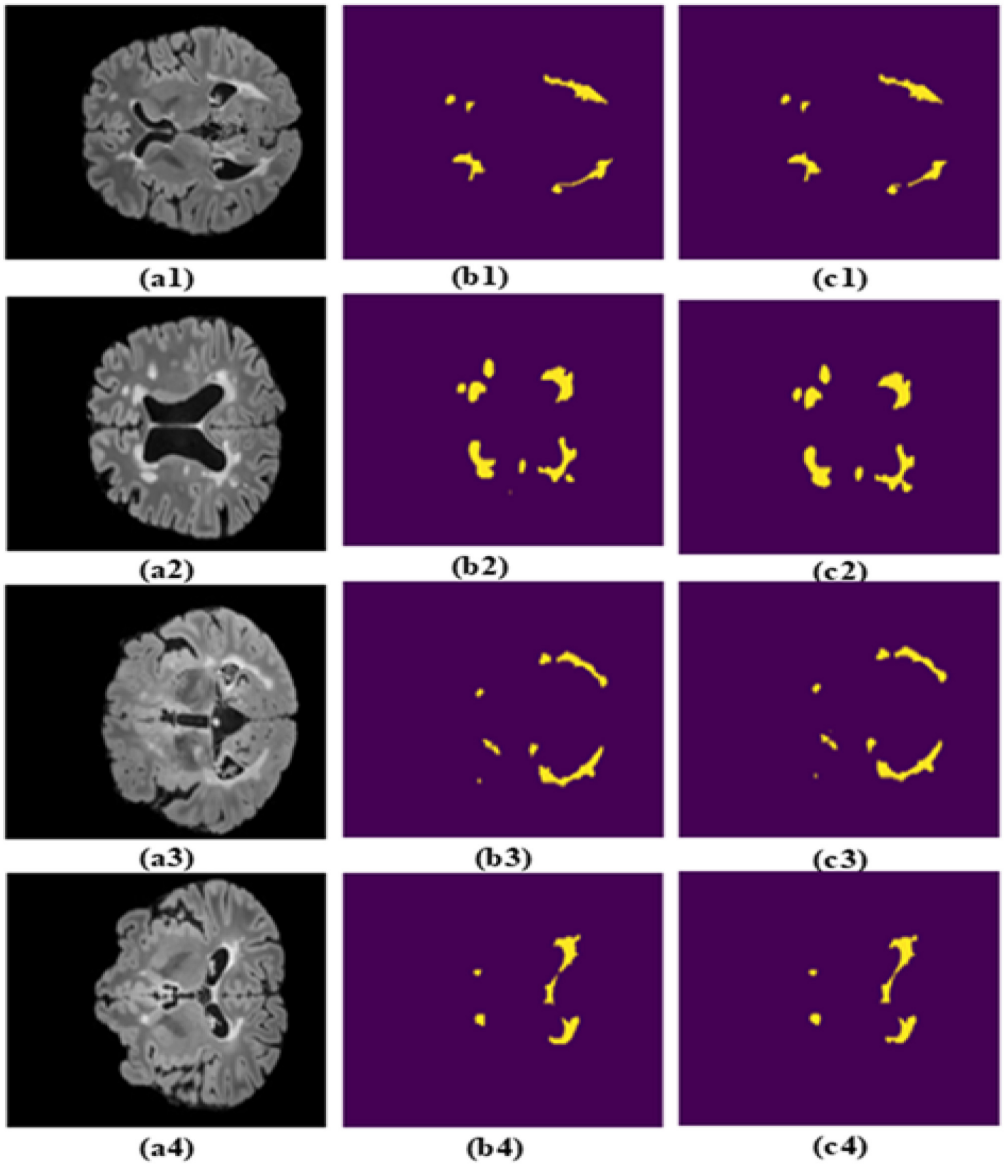
Results obtained on the MICCAI-2016 dataset. **(a1–a4)** Brain MRI image. **(b1–b4)** Corresponding ground truth image. **(c1–c4)** Segmentation obtained using MS-DASPNet.

The MS lesions segmented by the MS-DASPNet on the images of the MICCAI-2016 dataset are presented in Figures 11a1–a4 and are visualized in Figures 11c1–c4. The reference MS lesions are shown in Figures 11b1–b4. The experimental results on test data of the MICCAI-2016 dataset for different quantitative parameters are presented in Table 6. On the MICCAI 2016 dataset, MS-DASPNet achieves a Dice score of 0.8736 and a Jaccard index of 0.7982, indicating that the method used has segmented the MS lesion efficiently. The precision of 0.8746 and sensitivity of 0.8834 indicate that the model is performing accurately in detecting MS lesions while minimizing the FPR. The high sensitivity (0.8834) indicates that MS-DASPNet successfully identifies even subtle lesion regions in this particular case, which is critical in medical image analysis, where overlooking affected regions can lead to serious diagnostic implications.

#### 4.2.3 On MICCAI-2021 dataset

**Figure 12:**
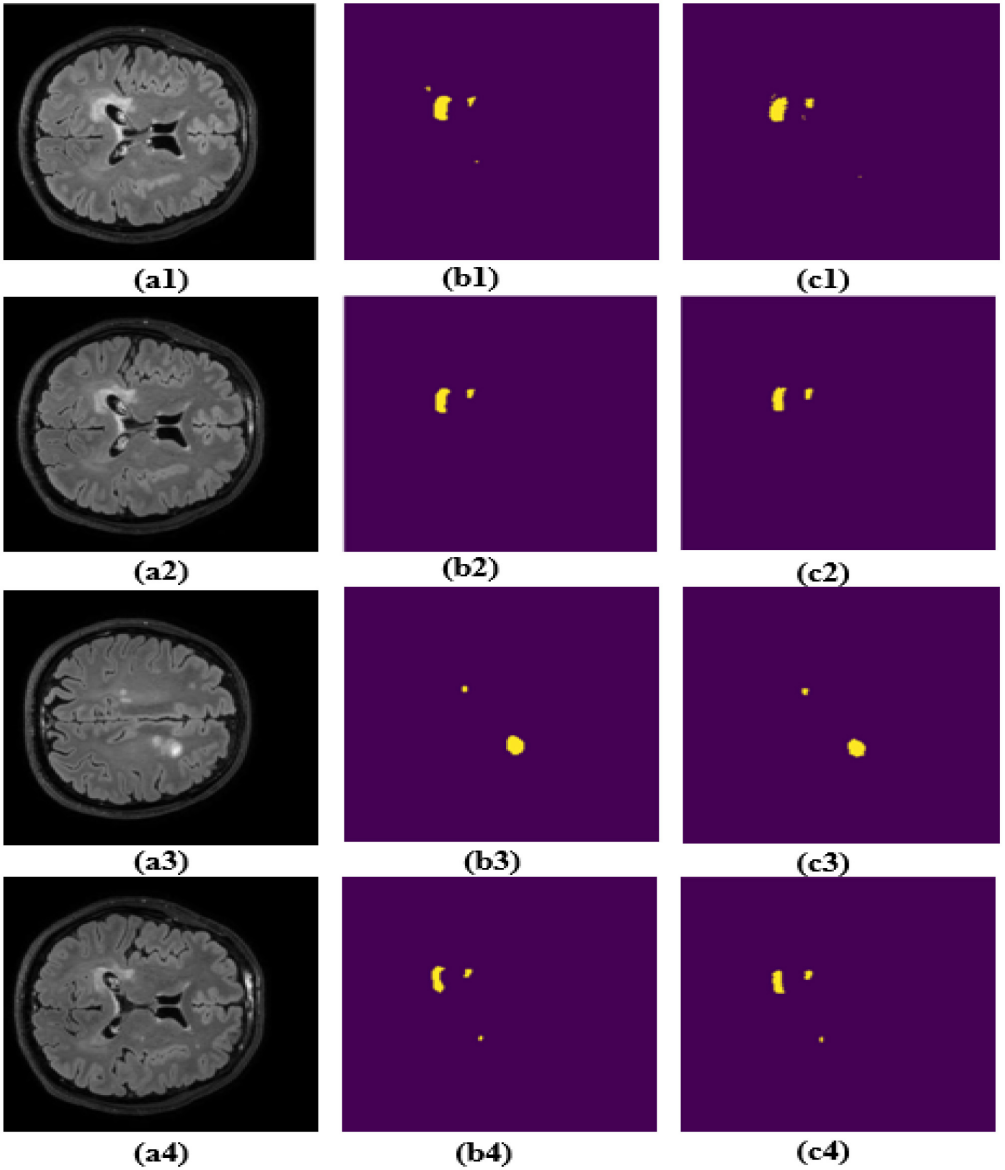
Segmentation results obtained MICCAI-2021 dataset. **(a1–a4)** Brain MRI image. **(b1–b4)** Corresponding ground truth image. **(c1–c4)** Segmentation obtained using MS-DASPNet.

The effectiveness of the proposed MS-DASPNet architecture on the MICCAI-2021 dataset is illustrated in Figure 12 and summarized in Table 6. The segmented MS lesion shown in Figures 12c1–c4 corresponds to images shown in Figure 12, which reveals that MS-DASPNet segments MS lesions from MRI scans efficiently. On the MICCAI 2021 dataset, MS-DASPNet maintains consistently high segmentation accuracy, showcasing its robustness in detecting MS lesions under varying imaging conditions. The model achieves a Dice coefficient of 0.8706 and a Jaccard index of 0.7719, indicating strong agreement with expert-labeled lesion masks. The high specificity (0.9996) and low FPR (0.0004) values highlight its excellent background discrimination capability, which is critical for accurate MS lesion segmentation.

#### 4.2.4 On Mendeley dataset

**Figure 13:**
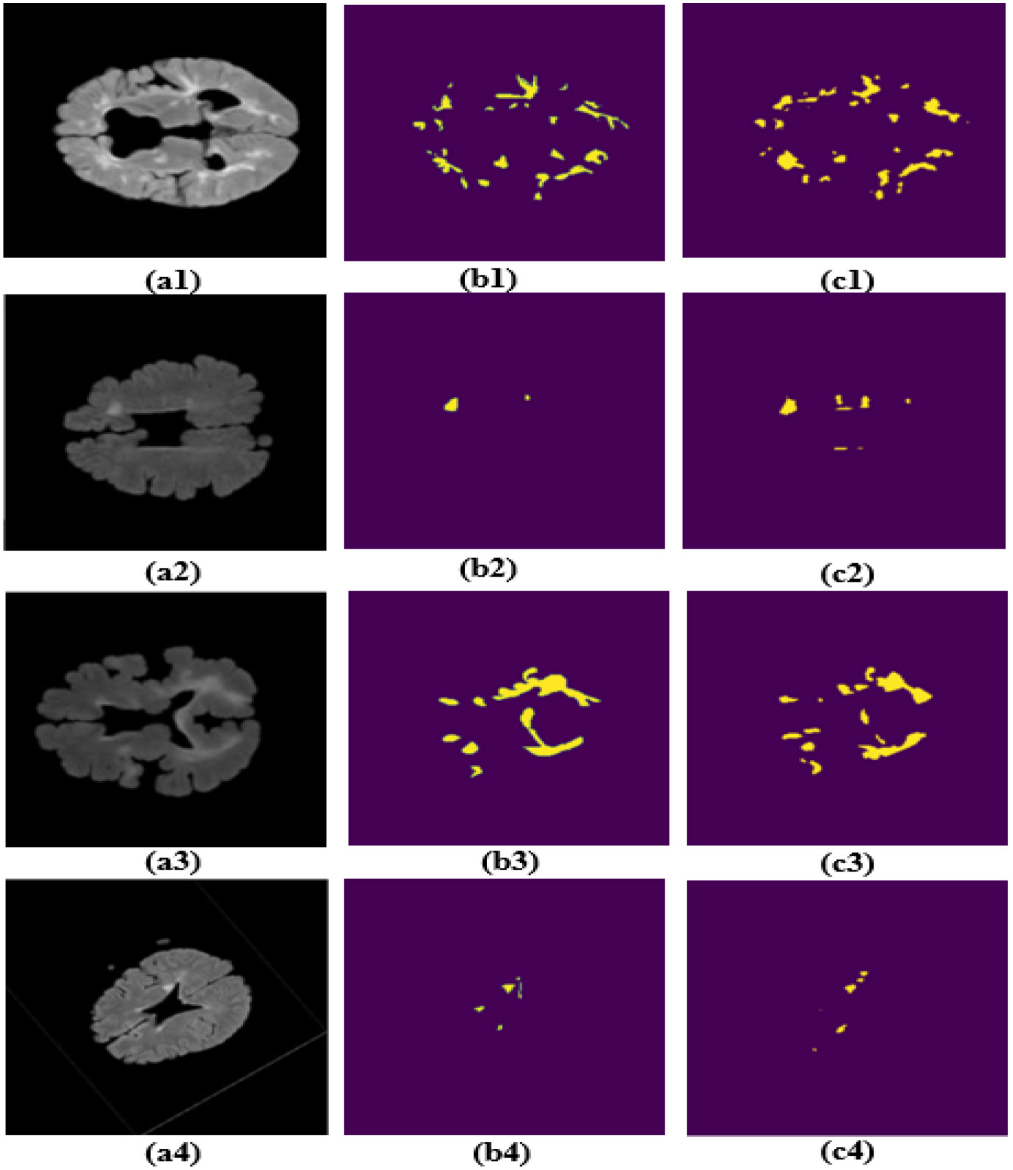
Segmentation Results obtained on Mendeley dataset. **(a1–a4)** Brain MRI image. **(b1–b4)** Corresponding ground truth image. **(c1–c4)** Segmentation obtained using MS-DASPNet.

The experimental results of the proposed MS-DASPNet model are presented in Figure 13 and evaluated on different evaluation metrics as presented in Table 6. In the Mendeley dataset, MS-DASPNet has performed comparatively poorly relative to other datasets, which indicates challenges in accurately identifying MS lesions. The model achieves a Dice coefficient of 0.5076 and a Jaccard index of 0.3519, which reflect its inability to handle the artifacts present in MRI Scans. Despite these limitations, the model maintains a high specificity (0.9985) and a low false-positive rate (0.0015), implying that it effectively distinguishes non-lesion regions and avoids false detections.

MS-DASPNet was evaluated on four publicly available FLAIR MRI datasets, which are highly efficient at emphasizing hyperintense white matter lesions that are characteristic of MS. The model demonstrated superior performance on MICCAI 2016 and MICCAI 2021, with Dice scores of 0.8736 and 0.8706, respectively, indicating strong spatial overlap with clinically annotated lesion masks. These results prove that the model is efficient in precisely demarcating periventricular and juxtacortical MS lesions, even in the presence of complex morphology. Sensitivities above 0.85 and FPRs below 0.0005 on these datasets indicate that MS-DASPNet can identify fine lesion patterns without generating false alarms in normal-appearing white matter.

On the ISBI 2015 dataset, the model achieved high precision (0.9252) and balanced Dice and recall scores, reflecting a consistent trade-off between detection and false-positive control. It was comparatively lower on the Mendeley dataset, with a Dice score of 0.5076 and a sensitivity of 0.4357, likely due to differences in lesion presentation or poor image quality. Notwithstanding this, the model consistently achieved a specificity of >0.998 in all datasets, affirming its reliability in suppressing non-lesion brain areas. In general, MS-DASPNet holds great promise for clinical utility in automated quantification of MS lesion load, assessment of disease progression, and tracking of treatment response.

## 5 Comparative analysis

In this section, the performance of the proposed MS-DASPNet is compared with well-known DL based architectures designed for the segmentation of MS lesions as presented in Tables 7–10. The Dice score is selected as a standard parameter for assessing the performance of MS lesion segmentation, as it addresses the issue of class imbalance. Generally, MS lesion regions are small compared to the background, and the accuracy metric can yield false results. However, the Dice score emphasizes the correct classification of lesion regions, avoids issues of background pixels, and is more sensitive to FPR.

**Table 7:** Comparative evaluation of the proposed method with state-of-the-art methods on ISBI-2015 dataset using dice coefficient.

**Method**	**Dice coefficient**
Unet (Ronneberger et al., 2015)	0.7785
DenseUNet (Cao et al., 2020)	**0.8808**
Attention-Unet (Oktay et al., 2018)	0.6126
Res-UNet (Zhang et al., 2018)	0.8084
Birenbaum and Greenspan (2016)	0.6271
Valverde et al. (2017a)	0.6294
Ansari et al. (2021)	0.6306
Azad et al. (2024)	0.7560
MS-DASPNet	0.8329

Bold means best.

### 5.1 On ISBI 2015 dataset

On the ISBI 2015 dataset, the performance of the proposed technique is matched with other DL architectures based on UNet and its variants, like UNet Ronneberger et al., (2015), DenseUNet Cao et al., (2020), Res-UNet Zhang et al., (2018), Attention-Unet Oktay et al., (2018), along with other DL architectures such as transformer-based Azad et al., (2024), patch-based, multi-view CNN Birenbaum and Greenspan, (2016), CNN with three Inception modules Ansari et al., (2021), and cascaded 3D CNN (Valverde et al., 2017a). A detailed comparison of the performance of the proposed method on the ISBI-2015 dataset is presented in Table 7. It has been observed that MS-DASPNet achieves a Dice score of 0.8329, outperforming well-established models such as UNet, Res-UNet, and Attention UNet. It also significantly surpasses CNN-based techniques like those proposed by (Birenbaum and Greenspan 2016), (Ansari et al. 2021), and (Valverde et al. 2017a), whose performance ranges from 0.62 to 0.63, partly due to their reliance on limited contextual understanding. Although on the ISBI-2015 dataset DenseUNet has obtained the highest Dice score of 0.8808, which is 5% higher than the proposed method, this is due to its increased computational complexity arising from the dense architecture. Among all the techniques presented in Table 7, the MS-DASPNet architecture has obtained the second-best and promising results in the segmentation of small, scattered lesions even in areas affected by low tissue contrast, intensity inhomogeneity, or partial volume effects—common challenges in brain MRI segmentation.

### 5.2 On MICCAI-2016 dataset

On the MICCAI-2016 Dataset, the performance of MS-DASPNet is matched on the Dice score and Sensitivity metric with various DL architectures UNet (Ronneberger et al., 2015), DenseUNet (Cao et al., 2020), Res-UNet Zhang et al., (2018), Attention-Unet Oktay et al., (2018), CNN-based architectures proposed by (Beaumont and Greenspan 2016), (Vera-Olmos et al. 2016), (Mahbod et al. 2016), and patch-wise DNN by (Kaur et al. 2024). Furthermore, the performance is compared with the traditional method based on Expectation-Maximization and graph-cut-based segmentation by (Beaumont et al. 2016b), rule-based techniques proposed by (Beaumont et al. 2016a), and intensified edge-based by (Knight and Khademi 2016). The detailed comparison is presented in the Table 8, and it has been observed that MS-DASPNet has the highest Dice Score of 0.8736, surpassing most benchmark DL models on Dice score, like UNet of 0.8635, DenseUNet of 0.8657, Attention-UNet of 0.8663, and Res-UNet of 0.8692. White's CNN architecture by (Kaur et al. 2024) is a close competitor to MS-DASPNet, achieving a Dice score of 0.8700. However, on the sensitivity metric, Res-UNet has achieved a slightly better result, 0.0009, than MS-DASPNet. MS-DASPNet exhibits a superior Dice Score, indicating a more balanced segmentation output with enhanced overlap accuracy. Whereas the traditional methods by (Beaumont et al. 2016b,a), (Knight and Khademi 2016) and (Vera-Olmos et al. 2016) have very low dice Scores, ranging from 0.5300 to 0.6000, reflecting their limited capability to generalize across lesion variations in FLAIR MRI due to reliance on handcrafted features, simple voxel-wise classifiers, or rule-based refinements.

**Table 8:** Comparative evaluation of the proposed method with state-of art methods on MICCAI-2016 dataset on dice score and sensitivity metric.

**Method and references**	**Dice score**	**Sensitivity**
Unet (Ronneberger et al., 2015)	0.8635	0.8233
DenseUNet (Cao et al., 2020)	0.8657	0.8442
Attention-Unet (Oktay et al., 2018)	0.8663	0.8521
Res-UNet (Zhang et al., 2018)	0.8692	**0.8843**
(Beaumont et al. 2016b)	0.5709	0.3584
(Beaumont et al. 2016a)	0.5300	0.5309
(Knight and Khademi 2016)	0.6000	–
(Mahbod et al. 2016)	0.6600	–
(Beaumont and Greenspan 2016)	0.5623	–
(Vera-Olmos et al. 2016)	0.5537	–
Kaur et al. (2024)	0.8700	–
MS-DASPNet method	**0.8736**	0.8834

Bold means best.

### 5.3 On MICCAI-2021 dataset

The comparative study of the proposed MS-DASPNet on the MICCAI-2021 Dataset is presented in Table 9 against several state-of-the-art segmentation approaches on dice-score. The MS-DASPNet method obtained the highest Dice Score of 0.8706, outperforming all counterparts. Although Res-UNet has a Dice Score of 0.8628, it showcases strong segmentation capability due to its residual connections. Moreover, DenseUNet, UNet, and Attention-UNet reported moderate Dice Scores of 0.7840, 0.6559, and 0.3914, respectively. Traditional and early methods, such as Basaran et al. (2022) and McKinley et al. (2021), had Dice Scores of 0.5100 and 0.6380, respectively, which reflect their limitations in handling the complex and heterogeneous nature of MS lesions in FLAIR MRI, which often exhibit high inter-patient variability and poor boundary definition. The proposed MS-DASPNet's strong performance on the MICCAI-2021 Dataset demonstrates its ability to effectively segment small, irregularly shaped lesions and maintain boundary integrity. Its integration of multi-scale feature aggregation via ASPP and attention-based contextual filtering enables it to achieve superior overlap with expert annotations.

**Table 9:** Comparative evaluation of the proposed MS-DASPNet method with state-of-the-art methods on the MICCAI-2021 dataset on dice score.

**Method**	**Dice score**
Unet Ronneberger et al., (2015)	0.6559
DenseUNet Cao et al., (2020)	0.7840
Attention-Unet (Oktay et al., 2018)	0.3914
Res-UNet Zhang et al., (2018)	0.8628
Basaran et al. (2022)	0.5100
McKinley et al. (2021)	0.6380
MS-DASPNet	**0.8706**

Bold means best.

### 5.4 Mendeley dataset

The comparative evaluation of the proposed MS-DASPNet is presented in Table 10 against several well-established DL models, UNet, DenseUNet, Attention-UNet, and Res-UNet. The best-performing method on this dataset is Attention-UNet, with a Dice Score of 0.6012, followed by DenseUNet and UNet. These results suggest that attention mechanisms and dense feature propagation provide tangible benefits in improving lesion detection and boundary delineation when applied to the Mendeley dataset. On the other hand, the proposed MS-DASPNet has a Dice score of 0.5076, which is lower than most of the evaluated DL models. While this result may appear suboptimal compared to the model's performance on other datasets (e.g., MICCAI-2016 and MICCAI-2021), it reflects the unique challenges presented by the Mendeley dataset. The lower Dice score on the Mendeley dataset can be attributed to greater variability in lesion appearance and increased lesion complexity. In particular, the dataset contains smaller and more fragmented lesion regions, which make accurate segmentation more challenging and increase sensitivity to minor boundary errors, thereby directly affecting the Dice metric.

**Table 10:** Comparative evaluation of the proposed MS-DASPNet with DL methods on the Mendeley dataset on dice coefficient.

**Method**	**Dice coefficient**
Unet (Ronneberger et al., 2015)	0.5533
DenseUNet (Cao et al., 2020)	0.5903
Attention-Unet (Oktay et al., 2018)	0.6012
Res-UNet (Zhang et al., 2018)	0.3704
MS-DASPNet	0.5076

**Figure 14:**
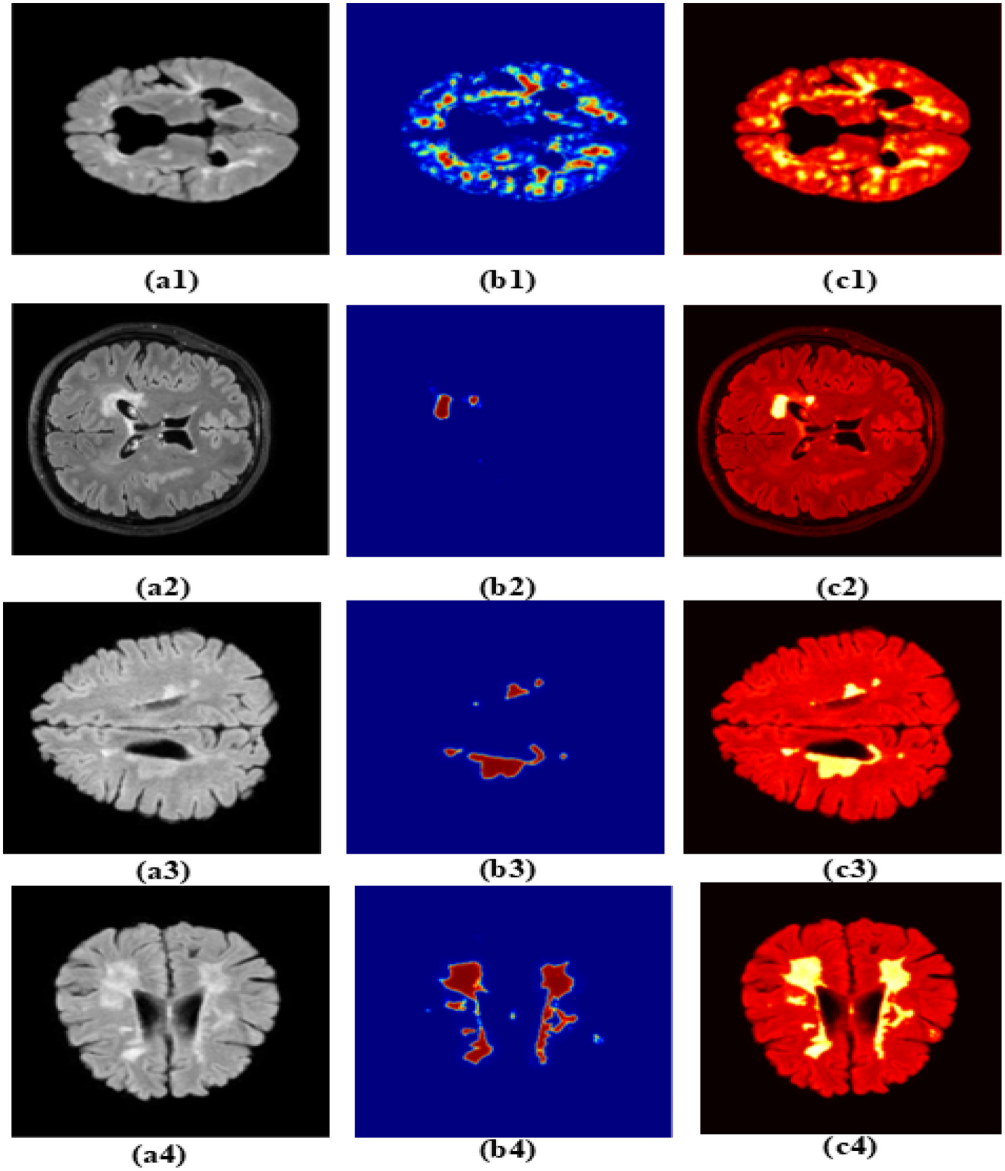
Attention map using MS-DASPNet. **(a1–a4)** Brain MRI image. **(b1–b4)** Attention map using GRAD-CAM. **(c1–c4)** GRAD-CAM overlay.

**Figure 15:**
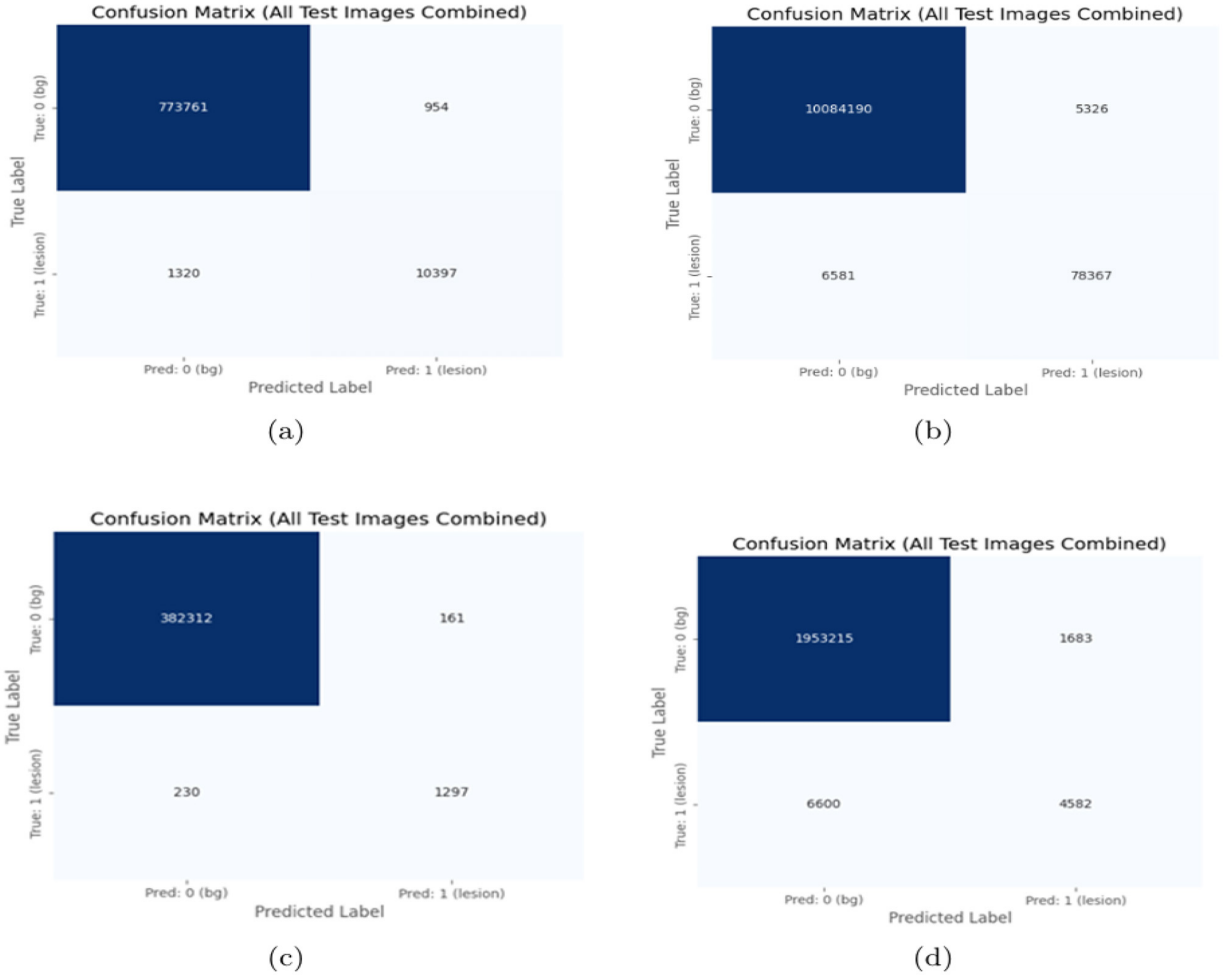
Confusion matrix of experimental results. **(a)** ISBI-2015 Dataset. **(b)** MICCAI-2016 Dataset, **(c)** On MICCAI-2021 dataset, and **(d)** Mendeley dataset.

The MS-DASPNet model integrates a VGG-16-based encoder, ASPP in the bottleneck for multi-level context learning, and dual-headed attention modules in the skip connections. This architectural synergy enables the model to effectively capture both coarse global structures and fine local details, leading to significantly improved segmentation performance. MS-DASPNet demonstrates superior performance on standardized datasets such as MICCAI-2016, MICCAI-2021, and ISBI-2015. Its moderate performance on the Mendeley dataset (Dice: 0.5076) highlights the importance of dataset diversity and domain adaptation in medical image segmentation tasks. The attention maps for selected representative images are illustrated in Figure 14 using the Grad-CAM technique. Specifically, Figures 14a1–a4 depict the original brain MRI slices, while Figures 14b1–b4 present the corresponding Grad-CAM heatmaps highlighting the regions that most strongly influence the model's predictions. Furthermore, subfigures Figures 14c1–c4 show the Grad-CAM overlays superimposed on the original MRI images, providing a clear visual interpretation of the model's focus. These visualizations demonstrate that the network effectively attends to clinically relevant lesion regions, thereby enhancing the interpretability and reliability of the proposed model. Overall, MS-DASPNet provides a well-balanced solution that not only enhances accuracy across various datasets but also maintains computational efficiency, making it a strong candidate for real-world clinical deployment in medical image segmentation tasks. The overall performance of the proposed method on different test datasets is presented as a confusion matrix in Figure 15.

## 6 Ablation study

An ablation study was performed to assess the robustness and generalization capability of the proposed MS-DASPNet under different training–testing configurations. The model was evaluated using three test split ratios, namely 10%, 15%, and 20%, and the corresponding quantitative results are reported in Tables 6, 11. Across all datasets, MS-DASPNet demonstrates consistent segmentation performance with only marginal variations in Dice and Jaccard scores as the test split changes. This behavior highlights the stability of the proposed architectural components and indicates that the model's performance is not overly sensitive to a particular data partition.

**Table 11:** Experimental results of MS-DASPNet on different quantitative parameters for 10%, 15%, and 20% test data.

**Dataset**	**Test split**	**Dice**	**Jaccard**	**Precision**	**Specificity**	**Sensitivity**	**FPR**
Mendeley	10%	0.5076	0.3519	0.7278	0.9985	0.4357	0.0015
	15%	0.2971	0.1993	0.4516	0.9991	0.2514	0.0009
	20%	0.2956	0.1991	0.4244	0.9991	0.2559	0.0009
ISBI 2015	10%	0.8329	0.7330	0.9252	0.9987	0.7804	0.0013
	15%	0.8638	0.7726	0.9477	0.9991	0.8067	0.0009
	20%	0.8608	0.7641	0.8319	0.9977	0.9048	0.0023
MICCAI 2016	10%	0.8736	0.7982	0.8746	0.9995	0.8834	0.0005
	15%	0.8803	0.8109	0.9086	0.9996	0.8626	0.0004
	20%	0.8743	0.8060	0.8896	0.9995	0.8659	0.0005
MICCAI 2021	10%	0.8706	0.7719	0.8943	0.9996	0.8503	0.0004
	15%	0.8042	0.6844	0.9257	0.9998	0.7280	0.0002
	20%	0.7783	0.6422	0.8881	0.9996	0.7062	0.0004

For benchmark datasets such as ISBI 2015 and MICCAI 2016, high Dice scores are maintained across all three test splits, accompanied by consistently high specificity and extremely low false positive rates, underscoring the model's ability to accurately suppress false lesion detections. Although slight performance fluctuations are observed when increasing the test split from 10% to 20%, these variations can be attributed to differences in lesion distribution and subject composition within the test sets, which is common in medical image segmentation tasks involving limited and imbalanced data. Overall, the ablation results confirm that integrating the proposed modules into MS-DASPNet yields robust and reliable lesion segmentation across varying data splits, thereby strengthening the validity of the reported performance claims.

## 7 Conclusion

This study presents **MS-DASPNet**, a UNet-based architecture designed for accurate lesion segmentation from brain MRI scans. MS-DASPNet employs a VGG-16 encoder, enabling robust and transferable low-level feature extraction. Including an ASPP bottleneck captures multiscale contextual information crucial for lesions of diverse sizes and shapes. Most notably, the dual-headed attention mechanism in skip connections enables the model to concentrate selectively on relevant spatial and channel-wise features, enhancing segmentation precision without incurring high computational costs. The approach begins with converting 3D brain MRI volumes into 2D slices, followed by skull removal using preprocessing techniques. Various image augmentation techniques were employed to mitigate overfitting caused by the limited dataset size and to enhance model generalization. Experiments were conducted on four publicly available datasets, and segmentation performance was evaluated using four metrics: Dice Score, precision, sensitivity, and Jaccard Index. The proposed MS-DASPNet model achieved the highest Dice Score of **0.8736**, outperforming several existing UNet variants. The proposed **MS-DASPNet** achieved a Dice Score of **0.8736**, outperforming several existing U-Net variants. Specifically, compared to the baseline **U-Net** model (Dice = 0.8635), MS-DASPNet achieved a **1.17%** improvement in Dice score, while surpassing **Attention U-Net** (Dice = 0.8663) by **0.84%** and **ResU-Net** (Dice = 0.8692) by **0.51%**. On the **MICCAI-2021** dataset, MS-DASPNet achieved a Dice Score of **0.8706**, reflecting a **0.82%** enhancement over the baseline and consistent improvement across all evaluation metrics.

Comparative analysis with state-of-the-art methods, including both traditional and DL-based techniques, revealed consistent improvements MS-DASPNet offers across different datasets. Specifically, on the MICCAI-2016 dataset, MS-DASPNet outperformed other architectures, including UNet variants, with the highest dice score of **0.8736**. Further evaluations on the MICCAI-2021 dataset show that MS-DASPNet outperformed other architectures, including UNet variants, achieving the highest Dice score of **0.8706**. In summary, the MS-DASPNet architecture demonstrates robust generalization, flexibility, and accuracy across multiple datasets, positioning it as a highly promising approach for medical image applications. In future work, k-fold cross-validation will be employed to assess further and enhance the model's robustness and generalization across diverse data distributions.

## Data Availability

Publicly available datasets were analyzed in this study. This study uses four types of datasets. (a) The ISBI-2015 dataset is available at: https://smart-stats-tools.org/lesion-challenge-2015 (b) The Mendeley dataset is available at: https://data.mendeley.com/datasets/8bctsm8jz7/1 (c) The MICCAI-2016 dataset is available by request from https://shanoir.irisa.fr/shanoir-ng/study/details/209 (d) The MICCAI-2021 dataset is available by request from https://shanoir.irisa.fr/shanoir-ng/study/details/208.
